# Supplementary material for: Symptoms in Health Care Workers during the COVID-19 Epidemic. A Cross-Sectional Survey
Source: Int J Environ Res Public Health. 2020 Jul 20;17(14):5218. doi: 10.3390/ijerph17145218 (PMC7400440; doi:10.3390/ijerph17145218)
Supplement: Supplementary file 1 [file ijerph-17-05218-s001.pdf]

Table S1. STROBE Statement—checklist of items that should be included in reports of observational studies.

	Item No	Recommendation	Check
**Title and abstract**	1	(*a*) Indicate the study's design with a commonly used term in the title or the abstract	*The type of study is indicated in the title*
		(*b*) Provide in the abstract an informative and balanced summary of what was done and what was found	*The abstract gives a summary of the study*
**Introduction**			
Background/rationale	2	Explain the scientific background and rationale for the investigation being reported	*Background and rationale are reported*
Objectives	3	State specific objectives, including any prespecified hypotheses	*Aims are detailed in the Introduction*
**Methods**			
Study design	4	Present key elements of study design early in the paper	*Key elements are reported*
Setting	5	Describe the setting, locations, and relevant dates, including periods of recruitment, exposure, follow-up, and data collection	*Setting is described*
Participants	6	(*a*) *Cohort study*—Give the eligibility criteria, and the sources and methods of selection of participants. Describe methods of follow-up<br>*Case-control study*—Give the eligibility criteria, and the sources and methods of case ascertainment and control selection. Give the rationale for the choice of cases and controls<br>*Cross-sectional study*—Give the eligibility criteria, and the sources and methods of selection of participants	*Cross-sectional study; eligibility criteria and methods of selection are detailed.*<br>*The comparison of HCWs with COVID-19 (cases) with a 2N sample of workers who had unprotected exposure and a 6N sample of HCWs without unprotected exposure and tested negative at RT-PCR test was done according to principles of case study, unconfounding and accuracy*
		(*b*) *Cohort study*—For matched studies, give matching criteria and number of exposed and unexposed<br>*Case-control study*—For matched studies, give matching criteria and the number of controls per case	
Variables	7	Clearly define all outcomes, exposures, predictors, potential confounders, and effect modifiers. Give diagnostic criteria, if applicable	*Predictors and outcome variables are described; possible confounders and modifiers are studied*
Data sources/ measurement	8*	For each variable of interest, give sources of data and details of methods of assessment (measurement). Describe comparability of assessment methods if there is more than one group	*Psychometric characteristics of questionnaires are reported.*<br>*Criteria for comparability of groups are reported.*
Bias	9	Describe any efforts to address potential	*Reporting bias deriving from*

		sources of bias	*incomplete answer was addressed removing these answers*
Study size	10	Explain how the study size was arrived at	*Sample size was evaluated with the formula suggested by Pocock:*<br>*N= f (α/2, β) * [p1 * (100- p1) + p2 * (100- p2)] / (p2 – p1)2*<br>*If we calculate the probability of finding a symptom in the CASE group and in the CONTROL group, we can calculate the size of the population, placing a significance level (alpha) at 5% and a power (1-beta) at 90%.*<br>*For a symptom such as anosmia, which has a prevalence of 42% in cases and 0.8% in controls, the minimum sample size involves 16 cases and as many controls, total = 32 observations.*<br>*For a symptom such as anxiety, which has a prevalence of 35% in CASES and 11% in CHECKS, the required dimensions are 60 per group, total 120 observations.*<br>*All calculations were carried out with the help of the automatic calculator: Sealed Envelope Ltd. 2012. Power calculator for binary outcome superiority trial. Available online at: https://www.sealedenvelope.com/power/binary-superiority/ [Access May 26, 2020].*
Quantitative variables	11	Explain how quantitative variables were handled in the analyses. If applicable, describe which groupings were chosen and why	*Method of handling variables was reported. The criteria for selecting groups were detailed.*
Statistical methods	12	(*a*) Describe all statistical methods, including those used to control for confounding	*Statistical methods were described*
		(*b*) Describe any methods used to examine subgroups and interactions	*Statistical methods were described*
		(*c*) Explain how missing data were addressed	*Cases with missing data were eliminated*
		(*d*) *Cohort study*—If applicable, explain how loss to follow-up was addressed<br>*Case-control study*—If applicable, explain how matching of cases and controls was addressed<br>*Cross-sectional study*—If applicable, describe	*Statistical methods were described*

analytical methods taking account of sampling strategy

(*e*) Describe any sensitivity analyses

e

**Results**			
Participants	13*	(a) Report numbers of individuals at each stage of study—eg numbers potentially eligible, examined for eligibility, confirmed eligible, included in the study, completing follow-up, and analysed	*Number of participants is reported*
		(b) Give reasons for non-participation at each stage	*Participation was voluntary.*<br>*Some workers stopped testing before the end and were eliminated for incomplete responses*
		(c) Consider use of a flow diagram	
Descriptive data	14*	(a) Give characteristics of study participants (eg demographic, clinical, social) and information on exposures and potential confounders	*Characteristics are reported and analysed*
		(b) Indicate number of participants with missing data for each variable of interest	*Answers with missing data were eliminated*
		(c) *Cohort study*—Summarise follow-up time (eg, average and total amount)	
Outcome data	15*	*Cohort study*—Report numbers of outcome events or summary measures over time	
		Case-control study—Report numbers in each exposure category, or summary measures of exposure	
		Cross-sectional study—Report numbers of outcome events or summary measures	*Numbers are reported*
Main results	16	(*a*) Give unadjusted estimates and, if applicable, confounder-adjusted estimates and their precision (eg, 95% confidence interval). Make clear which confounders were adjusted for and why they were included	*Unadjusted and adjusted estimates and their precision are reported*
		(*b*) Report category boundaries when continuous variables were categorized	*Age was categorized*
		(*c*) If relevant, consider translating estimates of relative risk into absolute risk for a meaningful time period	
Other analyses	17	Report other analyses done—eg analyses of subgroups and interactions, and sensitivity analyses	*All analyses done were reported*
**Discussion**			
Key results	18	Summarise key results with reference to study objectives	*Key results are summarized*
Limitations	19	Discuss limitations of the study, taking into account sources of potential bias or imprecision. Discuss both direction and magnitude of any potential bias	*Limitations of the study are discussed*

Interpretation	20	Give a cautious overall interpretation of results considering objectives, limitations, multiplicity of analyses, results from similar studies, and other relevant evidence	*The interpretation of the results was very cautious, given the cross-sectional nature of the study which does not allow to infer causality*
Generalisability	21	Discuss the generalisability (external validity) of the study results	*The generalisability was discussed*
**Other information**			
Funding	22	Give the source of funding and the role of the funders for the present study and, if applicable, for the original study on which the present article is based	*The study was not funded*

*Give information separately for cases and controls in case-control studies and, if applicable, for exposed and unexposed groups in cohort and cross-sectional studies.

Note: An Explanation and Elaboration article discusses each checklist item and gives methodological background and published examples of transparent reporting. The STROBE checklist is best used in conjunction with this article (freely available on the Web sites of PLoS Medicine at http://www.plosmedicine.org/, Annals of Internal Medicine at http://www.annals.org/, and Epidemiology at http://www.epidem.com/). Information on the STROBE Initiative is available at www.strobe-statement.org.
